# Intraosseous Delivery of Mesenchymal Stem Cells for the Treatment of Bone and Hematological Diseases

**DOI:** 10.3390/cimb46110752

**Published:** 2024-11-08

**Authors:** Mikhail Yu. Artamonov, Evgeniy L. Sokov

**Affiliations:** 1 Penn Medicine Princeton Health, Plainsboro, NJ 08536, USA; 2 Department of Algology and Rehabilitation, Peoples’ Friendship University, Moscow 117198, Russia; sokov_el@pfur.ru

**Keywords:** mesenchymal stem cells (MSC), intraosseous delivery, bone marrow niche, tissue regeneration, osteogenesis, immunomodulation, bone-related disorders, MSC retention, regenerative medicine

## Abstract

Mesenchymal stem cells are used most in regenerative medicine due to their capacities in differentiation and immune modulation. The intraosseous injection of MSC into the bone has been recommended because of expected outcomes for retention, bioavailability, and enhanced therapeutic efficacy, particularly in conditions involving the bone, such as osteoporosis and osteonecrosis. A review of the intraosseous delivery of mesenchymal stem cells in comparison with intravenous and intra-arterial delivery methods will be subjected to critical examination. This delivery mode fares better regarding paracrine signaling and immunomodulation attributes, which are the cornerstone of tissue regeneration and inflammation reduction. The local complications and technical challenges still apply with this method. This study was more focused on further research soon to be conducted to further elucidate long-term safety and efficacy of intraosseous mesenchymal stem cell therapy. Though much has been achieved with very impressive progress in this field, it is worth noting that more studies need to be put into place so that this technique can be established as a routine approach, especially with further research in biomaterials, gene therapy, and personalized medicine.

## 1. Introduction

The human body is composed of various types of cells that work together to form tissues and organs, each playing a vital role in overall health and maintenance. Among these are stem cells, which have unique characteristics of self-renewal and multilineage differentiation, playing an essential role in maintaining tissue homeostasis and repair [1]. Mesenchymal stem cells hold significant value in regenerative medicine due to their regenerative properties, including immunomodulatory capabilities and secretion of trophic factors. The intrinsic homing ability of mesenchymal stem cells enables them to migrate toward injury sites, promoting tissue repair and healing. This characteristic underlies the extensive use of MSCs in numerous clinical trials targeting a wide range of conditions [2]. This review aims to provide a comprehensive analysis of intraosseous (IO) delivery techniques as a therapeutic strategy for MSC application in treating bone diseases and hematological disorders. Although numerous studies explore various MSC delivery methods, a definitive consensus on an optimal approach is yet to be established. IO delivery techniques offer several advantages over systemic approaches, including higher cell retention, localized therapeutic effects, and improved engraftment within the bone marrow microenvironment. This review will detail the mechanisms of action associated with MSC and illustrate how their therapeutic potential can be enhanced through IO delivery [3]. Despite the promising outcomes observed with MSC therapies, the field faces several challenges, including determining optimal delivery methods and dosing. Variability in the route of administration, the biological context of the target condition, and characteristics across studies may influence the efficacy of MSC therapies [4]. Addressing these research challenges, emerging strategies such as cell pre-conditioning and priming are being developed to enhance MSC functionality post-delivery. This review aims to demonstrate the potential of intraosseous administration in optimizing MSC efficacy by overcoming the limitations associated with traditional delivery routes. It seeks to strengthen the rationale for IO delivery and emphasize its clinical significance. Additionally, we will discuss advancements in biomaterials and genetic modifications that may further bolster MSC therapy. The localized transplantation of MSCs, as opposed to systemic transplantation, affects the paracrine efficacy in synthesizing trophic factors. Specific paracrine signals are sent over small distances, resulting in localized effects, and the interaction between the local microenvironment of damaged host tissues and MSCs stimulates the generation of cytoprotective paracrine substances [5].

This review highlights the therapeutic role of intraosseous delivery techniques for MSCs, proposing it as a leading approach in the current landscape of regenerative medicine. Considering both scientific and clinical aspects, we have provided valuable information for researchers and clinicians interested in advancing MSC therapy for various disorders [6].

## 2. Mesenchymal Stem Cells

Human mesenchymal stem cells are a heterogeneous population of stromal stem cells that can be isolated from multiple adult organs. They can differentiate into mesodermal lineage cells, including adipocytes, osteocytes, chondrocytes, and cells from other embryonic lineages. Mesenchymal stem cells can engage with cells from both the innate and adaptive immune systems, modifying various effector functions. Following in vivo delivery, MSCs elicit peripheral tolerance and move to injured tissues, where they can suppress the release of pro-inflammatory cytokines and enhance the survival of damaged cells [7].

Furthermore, they are multipotent adult stem cells possessing multilineage differentiation capabilities and immunosuppressive characteristics, rendering MSCs an optimal choice for immunomodulation and regenerative medicine. MSC-related research and clinical studies have generated significant promise in numerous illnesses and tissue regeneration. It is essential to acknowledge that various significant potential issues have arisen from contemporary clinical trials, including (1) the ambiguous correlation between the phenotypic attributes and biological functions of MSCs; (2) the absence of clinical evidence to substantiate the long-term safety of MSCs; (3) the necessity for additional elucidation of the diverse mechanisms underlying MSC transplant actions in vivo; and (4) the inadequacy of comparability regarding MSC transplant efficacy. Consequently, MSC-based therapies cannot yet be regarded as standard clinical treatments [8,9].

Mesenchymal stem cells (MSCs) can transdifferentiate, enabling them to traverse lineage barriers and develop into neurons under specific experimental settings. Mesenchymal stem cells exhibit anti-proliferative, anti-inflammatory, and anti-apoptotic properties in neurons. Consequently, mesenchymal stem cells (MSCs) were evaluated in experimental autoimmune encephalomyelitis (EAE), an animal model of multiple sclerosis (MS), to assess their efficacy in modulating the pathogenic mechanisms of EAE for the development of effective MS therapies. The literature indicates that MSCs can suppress the activity of autoreactive T-cells in EAE, and this immunomodulation may confer neuroprotection. Moreover, MSCs can salvage neural cells by a method facilitated by soluble substances that create an optimal environment for neuronal regeneration, remyelination, and enhancement of cerebral blood flow [10].

It was shown that in early femoral head osteonecrosis, cell treatment could aid in bone development and remodeling. In a validated model of young pig femoral head osteonecrosis, this work investigated how intraosseous mesenchymal stem cells affect bone production and remodeling. Thirty-four 4-week-old Yorkshire pigs in all were used. All 31 animals in the trial suffered femoral head osteonecrosis in their right hip. One month following surgery, radiographs of the hip and pelvis validated femoral head osteonecrosis. Four animals dropped following surgery. Two groups were formed: A, mesenchymal-stem-cell-treated and B, saline-treated. Whereas the saline-treated group received five cc of physiological saline solution, the mesenchymal stem cell group received five cc of intraosseous injection of 10 × 10^6^ cells one-month post-surgery. To evaluate femoral head osteonecrosis, X-rays were obtained monthly for four months following surgery. One to three months after intraosseous injection, the animals died. After sacrifice, histology was done immediately to observe osteonecrosis and healing of femoral head tissue. Radiographs at the time of sacrifice revealed significant femoral head osteonecrosis and deformity in two of thirteen (15%) in the mesenchymal stem cell group and eleven of fourteen (78%) in the saline group. Histologically, the mesenchymal stem cell cohort showed flattening and reduced femoral head osteonecrosis. The saline group showed significant femoral head flattening and fibrovascular tissue replacing injured epiphyseal trabecular bone. Using intraosseous mesenchymal stem cells enhanced bone repair and remodeling in our young pig model of femoral head osteonecrosis. This finding implies that future research should investigate whether mesenchymal stem cells help in the healing of immature osteonecrosis in the femurs [11].

Mesenchymal stem cells (MSC) facilitate osteogenesis and represent a possible treatment for postmenopausal osteoporosis. The correlation between enhanced intraosseous microcirculation and augmented bone mass resulting from mesenchymal stem cells in postmenopausal osteoporosis remains ambiguous. As such, after thorough characterization and isolation of the primary MSCs, they were transplanted into ovariectomized mice. The transplantation of MSCs improved the trabecular count, trabecular bone volume relative to total volume, and trabecular bone mineral density in ovariectomized mice. Mice underwent femoral artery ligation to ascertain the function of mesenchymal stem cells in vascular healing. By using laser speckle flowmetry, microvascular perfused blood volume was measured, and both the vascularization of distal femoral trabecular bone and mid-femur cortical bone quality were analyzed to determine how much mesenchymal stem cells improve intraosseous-induced angiogenesis as well as increased osteoporosis in mice. Evidence for mesenchymal stem cells shows that they play a pivotal role in reconstituting appropriate cellular and humoral immune responses. They can also facilitate increased longevity with the successful inhibition of postmenopausal osteoporosis, connected to the restoration of microcirculation within the skeletal system [12]. Minimally modified mesenchymal stem cells (BMAC or SVF) are deemed safe and exhibit short-term advantages in treating knee osteoarthritis; nevertheless, further high-quality research is required to provide clinical practice recommendations [13].

## 3. Intraosseous Delivery Techniques

### 3.1. Manual Needle Insertion

**Figure 1:**
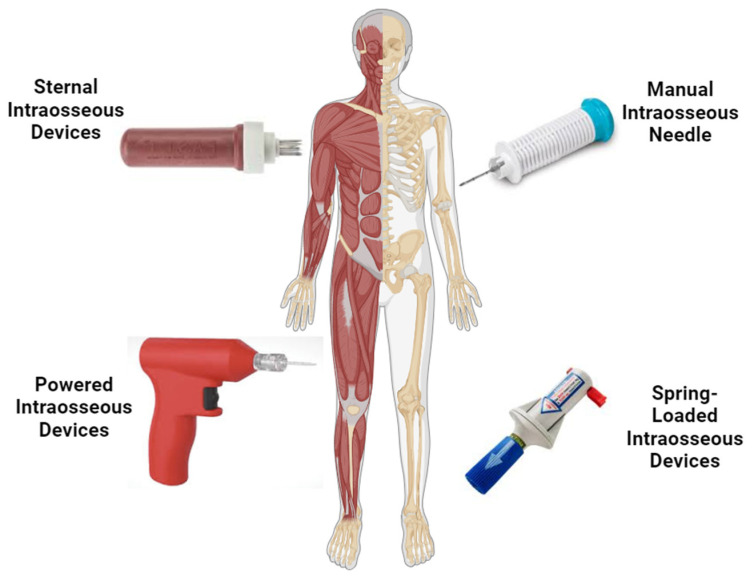
Illustrated different intraosseous delivery methods.

Resuscitation of critically injured trauma patients requires vascular access to replenish lost blood volume. This is optimally achieved by inserting a minimum of two large-bore intravenous catheters. These are generally situated in the antecubital veins. The veins may collapse in shock and traumatized patients. The implantation of an intravenous catheter could fail due to factors such as obesity, venous damage from recurrent intravenous drug use, and substantial extremity injuries [14]. Intraosseous (IO) access is another method to deliver fluid and medications directly into the bone marrow, primarily in the long bones, such as the tibia or humerus. Moreover, the sternum is a common intraosseous site, especially in a military context, as part of an expedient method of moving large-volume fluids into the central circulation [15]. This strategy is often used in emergencies, particularly in trauma settings were obtaining an IV line may be challenging and delayed. In patients with clinical cardiac arrest during active cardiopulmonary resuscitation, the American Heart Association and the International Liaison Committee on Resuscitation have recommended intraosseous access as one of the so-called “alternative pressure points” in which to deliver vasopressor drugs [16]. Intraosseous (IO) access provides reliable and rapid vascular access in critically ill patients when conventional intravenous access cannot be easily obtained. Its simplicity and low rate of complications have led to the widespread use of this technique in prehospital and hospital-based emergency care. Necessary to improve patient outcomes during critical scenarios is continued education and training on the appropriate use of IO devices and an understanding of their contraindications, as well as possible complications with their use. Figure 1 indicates some of the intraosseous devices explained below.

The metal Cook Dieckmann IO needle is usually shaped like a pyramid for use with adults and appears to be more pencil-shaped for children and infants. The insertion needle is just one part of the device, and it has both a sear and handle that we use to drive through the extra-cortical bone to find its interior. The catheter has a black mark 1 cm from the tip, which serves as the visual reference point for needle placement. The needle is inserted at a right angle to the joint. The insertion of this needle type is performed by applying pressure with a minor rotational motion to pierce the cortex of the adult patient [17]. BD^®^ Intraosseous Needles are the sole IO needles with an inbuilt passive needle tip safety mechanism to prevent needlestick accidents. The BD^®^ Intraosseous Vascular Access System safeguards healthcare professionals from accidental needlesticks, particularly prevalent in emergencies. They are aligned with INS Infusion Therapy Standards of Practice that recommend “the use of passive safety-engineered devices for needlestick injury prevention” and comply with ISO standards and guidance by the FDA for sharps injury prevention [18].

### 3.2. Powered Devices

Failed insertions prolong treatment times, diminish patient satisfaction, and substantially elevate the risk of consequences. More than 33% of adults and over 50% of children requiring a peripheral intravenous catheter (PIVC) upon hospital presentation are reported to have difficult venous access (DVA) [19]. These powered devices facilitate intraosseous access in adult and pediatric patients when intravenous access is challenging or unattainable in emergency, urgent, or medically essential situations for up to 24 h. They have a rechargeable battery that endures up to 12 times longer than the non-rechargeable Teleflex EZ-IO1, ensuring confidence and reliability [20].

### 3.3. Spring-Loaded Devices

Current guidelines advocate using intraosseous (IO) vascular access in adults when peripheral venous access is inaccessible. Most available evidence originates from children, animal models, cadaver research, or the prehospital environment. Therefore, we evaluated two intraosseous access devices in adult hospital resuscitations. We conducted a prospective, randomized clinical trial to compare two separate intraosseous access devices in adult subjects aged >18 years who required trauma or medical resuscitation by our Emergency Department staff when peripheral venous access had failed. Participants were each assigned randomly to either the spring-loaded bone injection gun (BIG) or the battery-powered EZ-IO. The assessment standards contained the first-attempt success rate, procedure duration, and complication rates. The study involved forty consecutive adults in cardiac arrest, where half were randomized to receive treatment with the BIG, and half received the EZ-IO device, which was used as a control group. The overall first-attempt success rate was 85%, with a mean procedure duration ± SD of 2.0 min ± 0.9. A comparison of the two devices revealed an 80% success rate on the first attempt with the BIG and 90% with EZ-IO, and a mean procedure time of 2.2 min ± 1.0 for the BIG versus 1.8 min ± 0.9 for EZ-IO. No statistically significant differences were observed between the two IO devices. There were no significant complications such as infection, extravasation, or hemorrhage. Intraosseous vascular access is a safe and reliable method of obtaining vascular access in adult emergency hospital patients requiring resuscitation. Additional research is warranted to establish the performance of different intraosseous devices.

Intraosseous access provides several advantages, including the rapid establishment of vascular access within minutes, which is essential in emergencies. Intraosseous access frequently proves more effective than peripheral intravenous attempts in patients with challenging venous access. Moreover, drugs and fluids delivered through intraosseous access are absorbed into the systemic circulation with pharmacokinetics comparable to those provided intravenously. Notwithstanding its efficacy, IO access is regarded as a provisional solution and should be supplanted with IV access at the earliest opportunity. Limitations include a brief duration, as intraosseous access is often advised for a maximum of 24 h because of the heightened risk of infection. Secondly, intraosseous devices and needles frequently incur higher costs than conventional intravenous catheters, although this expense may be warranted in emergencies. Furthermore, adequate IO access relies on the clinician’s procedural and instrument proficiency. Practical training is crucial to decrease the likelihood of problems [21].

## 4. Mechanisms of Mesenchymal Stem Cell Stimulation via Intraosseous Delivery

**Figure 2:**
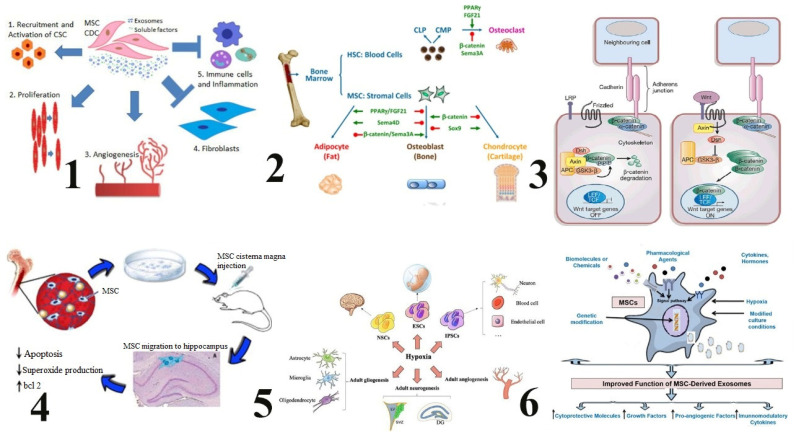
Mechanisms of mesenchymal stem cells.

Mesenchymal stem cells’ intraosseous delivery benefits the patient by directly targeting the bone marrow, thus optimizing MSC therapeutic potential to improve cell retention, engraftment, immunomodulation, and tissue repair, as demonstrated in Figure 2. Part 1). Exosome and soluble factor secreted by MSCs and CDCs contribute heart regenerationby promoting the recruitment of stem cells from cardiac origin, enhancing proliferation of cardiomyocytes proliferation and angiogenesis, and suppressing fibrosis and inflammation [22]. Part 2). This figure depicts differentiation of BMMSCs or HSCs into lineages in the context of bone and marrow with shown ligands, receptors, and signaling molecules that direct lineage allocation and fate. Common lymphoid and myeloid progenitors are labeled as CLP and CMP, respectively [23]. Part 3). Gene expression is regulated through the Wnt/β catenin pathway, controlling the stability of β-catenin. Although β catenin is degraded upon inactive Wnt signaling, in active signaling it stabilizes and allows entry into the nucleus to activate target genes transcription [22]. Part 4). Mesenchymal stem cells, cultured and injected directly into the cerebrospinal fluid of rodents, migrate to the hippocampus where they lessen the process of apoptosis; decrease superoxide production; and increase bcl-2 expression, making them neuroprotective [24]. Part 5). Hypoxia promotes stem cell self-renewal, differentiation, survival, and pluripotency in ESCs, NSCs, and iPSCs. Hypoxia also modulates adult neurogenesis, angiogenesis, and gliogenesis of DG, LV, and SVZ [25]. Part 6). Strategies include the introduction of biomolecules, pharmacological agents, cytokines or hormones, culture under hypoxia, and genetic modification to MSC-derived exosomes. Such modification steps enhance the content of exosomes; they are supplemented with these cytoprotective molecules, growth factors, and immunomodulatory agents for better therapeutic efficacy achievement [26].

### 4.1. Direct Access to the Bone Marrow Niche

Intraosseous delivery of mesenchymal stem cells exhibits prominent advantages by directly introducing the cells into their native bone marrow microenvironment, which is abundant in the growth factors, cytokines, and extracellular matrix responsible for supporting the survival, proliferation, and differentiation of MSCs [27]. This direct interaction has the advantage of improving integration, as MSCs can contact other cells such as osteoblasts (responsible for bone formation) and osteoclast cells (cells responsible for bone resorption). The bone marrow ECM acts as a scaffold for MSC engraftment and subsequently enhances the chances of successful grafting into the target tissue and proper functioning [28]. Furthermore, the cross-talk of MSC with various cell types within the bone marrow, such as hematopoietic stem cells and immune cells, creates a nurturing niche that confers their regenerative function via paracrine signaling [27,29]. Bone marrow MSCs are aided in their passage, survival rate, and proliferation, with direct contact from osteoblast cells, coupled with endothelial cells and immune cells. These interactions are thought to underlie the ability of MSCs to respond to biochemical and mechanical signals, which ultimately tunes their therapeutic activity [29].

### 4.2. Paracrine Mechanisms

The addition of intraosseously delivered mesenchymal stem cells (MSCs) to the bone marrow niche causes a paracrine action of signaling. These signaling pathways may aid in tissue regeneration, reduce inflammation and nerve injury, and enhance osteogenic differentiation [30]. MSCs implanted intraosseously within the bone marrow niche release the paracrine factors like vascular endothelial growth factor (VEGF), insulin-like growth factor((IGF), and hepatocyte growth factor (HGF) [31]. This enhances the process of angiogenesis, thereby returning vascular integrity and allowing regeneration in injured parts. VEGF is effective in stimulating angiogenesis, the formation of new blood vessels, which facilitates an adequate blood supply to damaged tissues and thereby supports tissue regeneration. HGF and IGF also play a role in tissue repair by causing a stimulation of cell proliferation and migration, predominantly within bone and adjacent tissues. MSCs produce anti-inflammatory cytokines, such as IL-10 and TGF-β, to down-regulate inflammatory molecules and therefore promote healing [32]. This vascular supply of the bone marrow adds to the paracrine capability of mesenchymal stem cells, with the result that they stimulate osteogenesis and angiogenesis by endogenous recruits as well as systemic engraftments, both towards a favorable therapeutic function. This is important for bone healing, particularly in a scenario of bone injury or degeneration [33].

### 4.3. Enhanced Bioavailability and Retention

Intraosseous delivery of mesenchymal stem cells has prominent benefits as compared to intravenous (IV) delivery, notably with respect to bioavailability and enhanced retention within the target area. Unlike IV delivery, intraosseous delivery avoids the pulmonary capillary bed, thus sending MSCs directly into the bone marrow, which allows a higher concentration of cells to reach the targeted site [34]. By circumventing the pulmonary system, intraosseous delivery also diminishes the hazard of systemic embolism. The use of IV administration leads to an increased risk of embolism in organs like the lungs. Therefore, more MSCs are sequestered and thus deprived of their therapeutic activity. In contrast, intraosseous delivery effectively minimizes such risk through improved MSC long-term retention within bone marrow, thereby positively impacting their bioavailability and overall therapeutic efficacy [35,36].

### 4.4. Stimulation of Endogenous Repair Mechanisms

Intraosseous administration of mesenchymal stem cells effectively triggers endogenous repair processes. These signals serve to activate an array of restorative pathways promoting tissue healing and regeneration. Mesenchymal stem cells are directly in contact with the extracellular matrix (ECM) as well as with bone tissues shortly after delivery [37,38]. Furthermore, the ECM acts as a support structure keeping mesenchymal stem cells functionally fit throughout tissue repair stages. Once activated by their interaction with the ECM, MSCs discharge an array of paracrine factors including cytokines and growth factors. These factors are essential for supporting angiogenesis (new blood vessel formation), tissue regeneration, and inflammation regulation. In the case of cytokines, they can recruit and activate other cells responsible for repairing at the site [39]. Growth factors such as VEGF are capable of improving the healing of damaged tissues by vascularization [40]. MSCs not only directly lead to tissue repair but also release paracrine factors that mobilize the resident stem cells within the bone marrow. This interaction bolsters the body’s natural innate regenerative capacity, enhancing the entire repair process.

### 4.5. Immunomodulatory Effects

Several studies have demonstrated the increased immunomodulatory capacity of mesenchymal stem cells that are delivered intraosseously, owing to interaction with host immune cells such as macrophages and T-cells. To this end, MSCs are responsible for releasing anti-inflammatory cytokines like interleukin-10 (IL-10) near a significant number of pro-inflammatory ones such as tumor necrosis factor-alpha (TNF-α), that account for lessening chronic inflammation and lead to favorable conditions for tissue repair [41]. The bone marrow’s immune-rich environment strengthens MSCs’ ability to modify the immune response. By avoiding systemic circulation, intraosseous delivery concentrates MSC activity within the bone marrow, augmenting interactions with immune cells. This technique causes MSC to release anti-inflammatory factors more efficiently, leading to reduced immune response and improved healing capability [42].

### 4.6. Paracrine Signaling and Microenvironmental Interactions

Paracrine interaction through the release of bioactive molecules due to their role in the bone marrow microenvironment is crucial for MSC activation and function. Intraosseous injection provides a local ultra-effective growth-factor-rich microenvironment including VEGF, IGF, and HGF necessary for activating MSC to initiate tissue repair, immune reprogramming, and angiogenesis [43]. After intraosseous delivery, MSCs release anti-inflammatory factors, TGF-β, IL-10 and VEGF, leading to inflammation reduction and resulting in tissue repair and regeneration together with induction of angiogenesis [44]. VEGF promotes the blood supply to damaged tissues, and HGF promotes cell proliferation and spread. The immunosuppressive activity of IL-10 promotes a microenvironment facilitating regeneration, whereas the growth factors secreted by MSCs favor osteogenic differentiation, augmenting bone formation and regeneration of marrow [45].

### 4.7. Hypoxic Environment and MSC Proliferation

Favorable properties of the bone marrow hypoxic environment mediated by hypoxia-inducible factor-1 alpha (HIF-1α) are also critical for governing MSC function. These variants interestingly increase MSC migration, proliferation, and differentiation ability, which can make them better candidates for tissue repair and regeneration [46]. Hypoxia keeps MSCs in a primitive form to be utilized for differentiation into different cell types wherever desired. Moreover, a hypoxic environment could induce the differentiation of MSCs into osteoblasts and thus adjuvant bone healing [47]. Delivering MSC directly to this hypoxic environment increases the efficacy of these cells for therapeutic applications by stimulating proliferation, maintaining multipotency, and promoting osteogenic differentiation [48].

### 4.8. Cell-to-Cell Interactions and Enhanced Engraftment

Mesenchymal stem cells (MSC) transplanted intraosseously can be directly adjacent to a diversity of bone marrow cell types (i.e., osteoblasts, endothelial and immune cells) essential for MSC survival, proliferation, and differentiation. Osteoblasts secrete both factors driving MSC differentiation into the bone and those that attract MSC migration to blood vessels, while underlying ES endothelial cells promote maintenance of local vascular cellularity [27]. In addition, immunomodulatory properties of MSCs and interactions with immune cells may affect homing to the injury site or the innate immunogenicity of MSCs. This enables more efficient MSC “homing” via respective signaling molecules and ECM components in the bone marrow, with resultant enhanced tissue repair efficiency, and reduces the potential for loss of MSCs during transit from circulation to target tissues when compared to systemic delivery. Therefore, this method increases the efficiency of MSC-based cell therapies [49].

### 4.9. Activation of the Wnt Signaling Pathway

MSCs are delivered to the bone marrow compartment, a Wnt-ligand-rich environment necessary for the osteogenic differentiation of MSCs, and this local activation of the Wnt pathway is an absolute requirement for bone formation and its repair, as explained in Figure 2. Activated signaling cascades and transcription factors generated by this pathway direct which genes are switched on or off that regulate the formation of bone (osteogenesis) [34]. In situ delivery promotes cell–cell and cell–matrix interactions, which are required for proper MSC response and bone repair. It promotes the expression of osteogenic growth factors like bone morphogenetic proteins (BMPs). The use of MSCs encapsulated in delivery vehicles as an alternative to intravenous administration can enhance MSC engraftment into bone pathologies [50].

## 5. Factors Influencing MSC Activation and Proliferation

Mesenchymal stem cells (MSC) are well-known for their regenerative and multipotent differentiation capacity. The activation and proliferation of these MSCs are modulated by a host of factors, which can be classified into soluble factors, physical factors, intrinsic cellular properties, therapeutic interventions, and other extrinsic factors. This insight is of paramount importance for the rational development of MSC-based therapies.

### 5.1. Soluble Factors

Growth factor cascades are critical for MSC propagation and viability. Molecular signals that respond to key growth factors, e.g., epidermal growth factor (EGF), fibroblast growth factor (FGF), and insulin-like growth factor 1 (IGFB-1), activate the downstream Ras-GTPase, Raf, and MEK, leading to the stimulation of the ERK/MAPK signaling pathway or PI3K/Akt pathway, resulting in MSC proliferation [51]. Vascular endothelial growth factor (VEGF) and platelet-derived growth factor (PDGF) are also reported to support the expansion of MSCs. Cytokines such as interleukin-1 (IL-1) and tumor necrosis factor-alpha (TNF-α) can activate MSCs and guide their migration to the injury locations in parallel with enhancing cell proliferation [34]. However, anti-inflammatory cytokines such as IL-10 may regulate MSCs in a better direction and increase their immunomodulation effect [29]. Critical for tissue location-specific repair processes, chemokines also direct MSC signals to specific sites of tissue [31].

### 5.2. Physical Factors

The environment in which MSCs typically reside is hypoxic. Due to hypoxia, the cells become more proliferative or less differentiated, which is believed to be a potential beneficial mechanism for repair and regeneration. Low oxygen affects the BM fate and proliferation of MSCs (but notSmPC) through HIF-1α (hypoxia-inducible factor 1 Alpha). Physical activity and tissue stress activate mechanical stimulation in MSCs that leads to differentiation. Mechanical loading and shear stress may enhance the expansion of MSCs, which could be advantageous for tissue engineering purposes [52]. Logically, since ECM components serve as a structural support for MSCs and contain signaling cues, changes in cell behavior can be directed. The stiffness and the composition of the ECM, which contains proteins like collagen and laminin, may influence MSC adhesion, proliferation, and differentiation [53,54].

### 5.3. Intrinsic Cellular Properties

**Figure 3:**
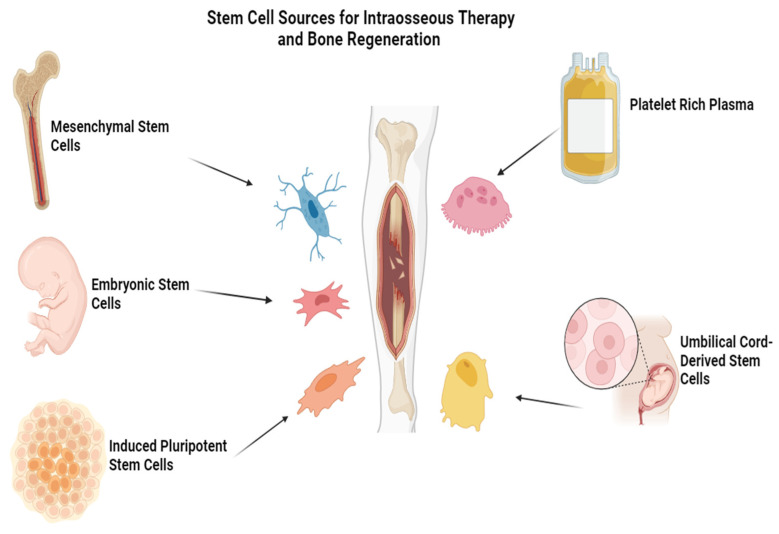
Sources of stem cells for bone regeneration.

The origin of the mesenchymal stem cells (MSC) used is critical. The most common sources of stem cells are bone marrow, adipose tissue, umbilical cord blood, and placenta, as shown in Figure 3, which differ hugely in their proliferative and differentiation potential. Therefore, Luger et al. emphasized that different MSC types possess specific features which modulate their differentiation potential and wound-regenerative abilities. Mesenchymal stem cells (MSC) isolated from aged individuals or people with diseases/dispositions often display decreased proliferative and regenerative capacity, in contrast to those obtained from younger and healthier donors, due to age-dependent alterations or premorbid states affecting their biological activity [55]. Modulations of DNA methylation and histone modification are found to direct MSC behavior by regulating gene expression that involves proliferation and differentiation [27].

### 5.4. Therapeutic Interventions

Growth factor supplementation involves the addition of specific growth factors to the culture media of mesenchymal stem cells (MSC) to enhance their proliferation and therapeutic efficacy. They promote cell division, enhance survival, and provide cues for MSC differentiation into various lineages through the stipulation of signaling pathways to drive differentiation efficiently in specific subsets of somatic stem cells, resulting in greater therapeutic efficacy [27]. Microcarriers are modulated three-dimensional structures resembling the nature of the ECM, providing mechanical and biological signals to MSCs. Enhanced by a 3D environment that supports cell proliferation, differentiation, and multi-tissue production through the presentation of multiple bioactive cues—such as growth factors as well as mechanical stimuli—scaffolds may help to regenerate and repair tissues when seeded with MSCs [52]. Gene editing tools such as CRISPR-Cas9 provide an opportunity to generate specific changes in the gene aspects of MSCs, and subsequently enhance their capacity. These technologies aim to tailor MSC differentiation skews toward functionally desired cell types, or improve the proliferation of potentially therapeutic MSCs [53].

Extrinsic factors influencing MSC delivery include the delivery route, host immune response, local microenvironment, and the specific characteristics of the targeted tissue or organ. The delivery method significantly affects MSC survival, retention, and distribution, with routes such as intravenous, intraosseous, or local injection each offering distinct outcomes. Immune rejection poses a potential barrier to MSC efficacy, as the host immune system may detect and clear the cells before they exert therapeutic effects. Additionally, the local microenvironment characterized by factors such as inflammatory signals, oxygen levels, and nutrient availability can impact MSC survival. To enhance MSC engraftment and functionality, several strategies are frequently employed, including biomaterial scaffolds, preconditioning, and MSC encapsulation in preparations designed to improve cell survival and integration within host tissue.

## 6. Intraosseous vs. Other MSC Delivery Mechanisms

Ease of access for the existing mesenchymal stem cells (MSCs) is rarely approved in several clinical applications owing to its broad differentiation potential, immune modulation, and employing a delivery tool that significantly influences therapeutic outcomes. This review concentrates on the approach of intraosseous administration of MSC, comparing it with intravenous (IV), intra-arterial, local injection, intrarectal, intramuscular, intranasal, and intracoronary routes, as shown in Table 1.

### 6.1. Intraosseous Delivery

One way of delivering mesenchymal stem cells (MSC) is to inject them directly into the bone marrow cavity (or intraosseously), with the intention of providing an in vivo homeostatic environment with physiological factors conducive to cell engraftment and function. This method is very effective, especially in bone-related disorders, because it activates the Wnt signaling pathway with the essential components for MSC-mediated osteogenesis and bone regeneration [57]. Wnt ligands can activate intracellular signaling cascades in the bone marrow that favor the differentiation of MSC into osteoblasts and the expression of bone morphogenetic proteins (BMPs), which are primarily responsible for bone repair. Intraosseously injected, MSCs have improved ability to home and engraft in their local stem cell niche, establishing stronger paracrine loops with local cells/growth factors needed for the maintenance of functionality as well as survival, proliferation, and differentiation [50]. This method not only improves the bioavailability and efficacy of MSCs but also enhances the local microenvironment around injury damage to sustain tissue repair. The bone marrow stroma is a permissive milieu for MSC survival and efficacy via growth signals, oxygen tension regulation, and nutrient availability, making this site ideal for delivery of MSCs. Nevertheless, the idea of introducing IO is controversial because it is more invasive than noninvasive methods, and thus it may cause discomfort or complications such as infection, local hemorrhage, or pain at the injection site. Due to the non-applicability of this method for systemic or localized delivery, and that it may achieve a limited efficacious response in extra-skeletal-system bone tissue injuries or organs, it is less practical for other situations like fractures. It is mostly suggested for used only in bone related diseases such as osteoporosis, fractures, or marrow disorders.

### 6.2. Intravenous Delivery

Mesenchymal stem cells (MSCs) can be directly injected into the body through an intravenous (IV) delivery, whereby the MSCs are released into the bloodstream and circulate within it while providing access to all tissues and organs [58]. Although advantageous in terms of systemic homing of MSCs to tackle multiple site-associated problems in vivo, displacement can be problematic due to the propensity for clotting that can occur during lung entrapment or occlusion in renal tubules, thus resulting in a partially effective means of pressurized delivery. Intraosseous delivery, on the other hand, is a less invasive method of therapeutic intervention that permits direct application into bone marrow with minimal perturbation of surrounding tissue [38]. This feature of the method makes it ideal for situations where a large number of organs are involved, or where the disease is systemic and concentrated medication to one area is not possible. On the other hand, systemic administration, such as IV injection of MSCs, is hindered by low engraftment rates owing to rapid entrapment in organs like the lung, inhibiting their ability to efficiently home to and treat target tissues. The effects of therapy may be inhibited if the agents are disseminated too widely, leading to lower concentrations at the site of interest, and low impact. In general, IV delivery might deliver the protein more entirely to the body, but intraosseous delivery offers a targeted method of administration which may be better suited for some applications, particularly those related to bone [57].

### 6.3. Intra-Arterial Delivery

Intra-arterial delivery provides targeted treatment and a higher concentration of the delivered mesenchymal stem cells (MSCs) in specific organs or tissues, leading to more potent therapeutic effects and less systemic circulation, which may be beneficial for the efficacy and safety of therapy. This approach has the advantage of a higher concentration of MSCs to be delivered directly into target sites, hence ensuring better treatment response and outcomes, the MSCs not being diluted as is the case in IV methods [59]. Intraosseous delivery, an administration route that provides local effects similar to IT injection, increases the probability of MSCs remaining and integrating at the intended site in BM, leading to closer interactions with the resident microenvironment, hence proper action and therapeutic outcomes. Intra-arterial delivery, on the other hand, is associated with an incremental emboli risk, especially within the small conduits, making it seem to be a bold step in terms of safety. In addition, the intraosseous method is comparatively complex to handle technically; it requires specific techniques and equipment, which may account for some procedural skill background effect in equipment maintenance [60,61].

### 6.4. Local Injection

Local MSC injection refers to the direct delivery of MSCs to the target site with an achieved rapid internality effect and the immediate establishment of regeneration, as these will have the best impact on the wound healing process. This approach can magnify the therapeutic effects of IU injection by packing all needed cells at a single local site and meanwhile avoid off-target side effects, thus enhancing efficiency and eliminating side effects due to systemic delivery [62]. Scaffold-based MSC delivery has gradually become a research focus because accelerating MSC interactions with injured tissues can enhance the efficacy of MSCs, which would be potentially beneficial for improving local MSC concentration compared to systemic methods [58]. Such a local strategy allows increased target site retention of a critical percentage of MSCs, ultimately leading to bone regeneration and repair. Invasive methods such as intraosseous delivery are, however, painful and potentially risk infection. Moreover, the volume of MSCs that can be delivered intraosseously is restricted. This presents a challenge in treating larger injury sites that may necessitate more cells for tissue repair or regeneration. This may result in insufficient MSCs transplanted, especially for vast damaged areas, to give a full therapeutic effect [58].

### 6.5. Emerging Strategies

Encapsulating therapeutic cells such as MSCs within biocompatible scaffolds (biomaterial-based delivery) is an established approach to provide controlled release and immune isolation, which might offer improved therapeutic outcomes [63]. This approach employs biocompatible components to establish an MSC-friendly microenvironment and tune it physically, chemically, structurally, and functionally based on the desired control of the cell release profile in time. It also reduces immune rejection after administration to enhance treatment efficacy. Furthermore, microneedle arrays enable the exact administration of MSCs or therapeutic agents to superficial tissues for local treatment, allowing at the same time an improvement in overall efficacy targeted at well-defined areas [64]. A non-invasive strategy involves intrarectal delivery, by which MSCs are provided through the rectum to allow them to migrate to the injury site without using more invasive procedures. This method is convenient to perform and requires no expensive instruments, providing an affordable means for MSC preparation. Intrarectal delivery is only suitable for conditions in which there is direct access to the rectum and so may be unsuitable in more general circumstances [63].

### 6.6. Intramuscular Delivery

IM-unmediated injection of MSCs enables direct incorporation and local muscle regeneration, with MSC serving either as myocytes or providing paracrine beneficial for bodily repair and regeneration [65]. This approach imparts a regional bias to MSCs and allows their effective muscle regeneration. The nutrients in the hematopoietic marrow provide an advantage for MSCs that are injected with intraosseous methods, which helps them survive longer and function better. Even the secretome, cytokines, growth factors, and exosomes may be released into the bloodstream, with the potential to have systemic effects in addition to local ones. Nevertheless, intramuscular injection is an invasive method in which the needle punctures muscle tissue, which may cause the patient to suffer. Therefore, despite driving muscle regeneration, such an approach raises concerns about invasiveness and convenience as opposed to intramuscular delivery [66].

### 6.7. Intranasal Delivery

Intranasal administration is a non-invasive approach to deliver mesenchymal stromal cells (MSCs) using the accessible nasal route, directly to the brain. This strategy benefits from the direct pathway of nasal mucosa to the central nervous system, allowing MSCs to reach the brain without the need for crossing the blood–brain barrier, which is a common obstacle faced by several therapeutic agents [67]. This approach enables the targeted delivery of therapeutic agents directly to the brain via the nasal route, avoiding invasive procedures. By facilitating direct transport through the nasal passage, this method is suited to chronic therapy and localized treatment. Its application in drug delivery can address severe neurological conditions, as it crosses the blood–brain barrier and predominantly affects central nervous system indicators. This technique has been widely used in brain disease research, allowing MSCs to reach the central nervous system bypassing the blood–brain barrier [43,68].

### 6.8. Intrathecal Delivery

Intrathecal delivery is one of the newest delivery procedures, in which mesenchymal stem cells (MSCs) are injected directly into the cerebrospinal fluid that bathes the spinal cord and brain. This way, MSCs will cross the BBB and move to different areas of cerebrospinal fluid and disperse into the spinal canal and brain, enabling them a high possibility of reaching the central nervous system ailment process [69]. It provides an alternative non-invasive approach for detecting and treating neurological disorders, instead of invasive methods that are often very complicated and dangerous [70]. Intrathecal delivery involves direct drug treatment to the spinal canal, and it might be an invasive method since it needs a spinal injection, which could evoke a painful sensation [69,71].

### 6.9. Intracoronary Delivery

Alternatively, mesenchymal stem cells (MSCs) are administered directly into great coronary vessels using standard coronary artery catheters. This is particularly helpful in treating heart disorders like heart attacks (myocardial infarctions or MI), where the stem cells are delivered directly to the dead part of the heart muscle, enhancing tissue repair and augmenting long-term structural and functional recovery of the injured myocardium [61]. This approach increases the engraftment rates of stem cells in the infarcted myocardium, resulting in greater attachment of stem cells to the injured tissue and thereby possibly enhancing new viable tissue genesis. Delivery via an intracoronary approach is associated with a risk of occlusion or damage to coronary vessels. This restricted blood flow may slow the flow of vital oxygen and other nutrients to the heart, which in turn can affect how well the heart performs [38,72]. Ideal conditions of MSC culture, including a temperature of 37 °C and CO percentage of 5%, significantly determine their viability and functionality. Variations in the present condition do alter cell growth to a significant extent and affect changes in differentiation potential, and thus have a ripple effect on the entire therapeutic efficiency [73,74]. This will involve the incubation duration and the number of passages for MSCs to be in their most stem-like state for their regenerative capacity. Long-term culture duration or multiple passages may cause cellular senescence as well as loss of potential therapeutic benefits [75]. Therefore, the culture medium for MSCs must be formulated carefully. The basal medium, supplemented with fetal bovine serum or human platelet lysate, is usually supplemented by essential growth factors like fibroblast growth factor or epidermal growth factor [76]. The composition of the medium has a direct influence on the proliferation, differentiation, and immunomodulatory properties of MSCs [77]. MSCs can be grown on various types of platforms, from classical 2D surfaces like tissue culture plastic to more sophisticated 3D scaffolds and hydrogels. These types of systems can function more closely in the in vivo environment, which might have beneficial effects on MSC functionality and therapeutic effectiveness [78,79].Cryopreservation is yet another important step used before cell transplantation, that is, freezing MSCs for long-term storage. Cryoprotectants used (e.g., dimethyl sulfoxide, DMSO) as well as freezing rates, and whether they use controlled rate freezing or direct freezing will considerably affect the viability and functionality of cells post-thawing. Inappropriately preserved cells suffer damage and decreased efficacy in therapeutic applications [80,81].

## 7. Clinical Applications

Mesenchymal stem cells (MSCs) are of utmost significance among bone marrow-derived stem cells (BMSC) for orthopedics, due to the fact that they can differentiate into osteoblasts and chondrocytes, bone- and cartilage-forming cells, respectively. Simply the possibility alone of injecting MSCs by intraosseous injection offers the advantage of immediate access to their normal location and thereby enhances homing, survival, and integration of these cells at the injury site. Because this direct delivery approach effectively promotes differentiation of MSCs into osteoblasts and chondrocytes—key cell types for efficient bone and cartilage repair/regeneration in a variety of bone/cartilage disorders [82]—this technique not only helps improve bone regeneration and decrease inflammation but also restrains joint degeneration, thereby providing a potential candidate for conservative bedside therapies [60]. Intraosseous delivery prevents the need for additional treatments since it is conducive to better MSC survival and incites targeted tissue repair, being more oriented and fruitful than systemic MSC delivery [71]. Concurrently, intraosseous delivery of MSCs is a potential strategy to treat multiple types of orthopedic diseases by improving MSC survival and promoting tissue repair and regeneration. It provides a safe and effective treatment option for a large number of orthopedic conditions [52].

### 7.1. Clinical Applications of Mesenchymal Stem Cells (MSC) via Intraosseous Delivery in Hematological Disorder

By sending MSCs to the bone marrow where the cells originate, this approach supports mobilization of endogenous HSCs into circulation, which is important in BM disorders with inadequate release of hematopoietic stem cells (HSC) by the bone marrow [29]. The intraosseous delivery of HSCs into the BM cavity of patients with bone marrow failure, such as aplastic anemia, not only allows for the creation of a supportive milieu for hematopoiesis but also enhances the regeneration of HSCs [83]. In addition, MSCs enhance the homing of transplanted HSCs and reduce GvHD while facilitating hematopoietic reconstitution after transplantation. Clinical trials found that some of these strategies offer potential benefits, such as hematopoietic recovery. Developed gene therapy has facilitated the extended use of MSCs in hemophilia A, particularly through intraosseous lentiviral vector administration. Nonetheless, the influence of MSCs on tumors is complicated, as they are known to exert either tumor-promoting or -suppressive effects based on different parameters. Thus, intraosseous MSCs display promise for hematological disorder treatment, but additional studies are required to improve the intervention and enhance clinical results [84].

### 7.2. Clinical Applications of Mesenchymal Stem Cells (MSC) in Systematic Diseases

Mesenchymal stem cells (MSC) have been increasingly recognized as regenerative and immunomodulatory agents for multiple systemic disorders. Clinical applications of MSCs in orthopedic conditions, hematological disorders, and systematic diseases are well demonstrated. They protect the heart by engrafting into cardiovascular and vascular cells and secreting paracrine factors to decrease fibrosis and promote function. MSCs secrete angiogenic factors that help to form new blood vessels and promote tissue healing, as is the case with pathological conditions like peripheral artery disease (PAD) [36]. Furthermore, in the case of autoimmune conditions, including systemic lupus erythematosus (SLE), MSCs could control immune responses and rheumatoid arthritis (RA) by reducing inflammation and destruction of joints. MSCs can contribute to remyelination and neuroprotection, which may be beneficial in neurological disorders such as multiple sclerosis. MSCs ameliorate renal dysfunction through their anti-inflammatory and regenerative properties in chronic kidney disease. In this condition, they can then become hepatocyte-like cells in liver disease environments to help ameliorate hepatic dysfunction and reduce fibrosis. MSCs play a great role in HSCT with their properties of immunomodulation and improvement of health outcomes. Similarly, MSCs were suggested to contribute to bone and cartilage repair and treatment of osteogenesis imperfecta as well as osteoarthritis, as discussed in much research. MSCs additionally drive macrophages and endothelial cells to aid stem tissue repair in wound healing [82]. MSCs offer a multipotent therapeutic strategy in many disease conditions, and further studies are required to develop treatment regimens with better safety profiles as time passes [85]. The clinical applications of Intraosseous delivery methods in orthopedic conditions, hematological disorders, and systematic diseases are demonstrated in Table 2.

## 8. Recent Advances and Future Directions

Stem cells are receiving increasing attention in regenerative medicine due to their ability of lineage differentiation and immunoregulatory properties, especially mesenchymal stem cells (MSCs). The use of intraosseous delivery systems is instrumental in addressing bone-related diseases through novel biomaterials including hydrogels and bioceramics to augment MSC retention and incorporation into bone tissue [86]. Future studies will include new strategies (such as magnetic targeting and exosome-based delivery systems) for better MSC localization and therapeutic effect. Over the past decade, it has become apparent that recent advances in bone substitute materials, such as biodegradable scaffolds made of polylactic acid (PLA) and polyglycolic acid (PGA), strongly enhance MSC growth and differentiation during bone healing [87]. Furthermore, bioactive coatings, hydrogel systems, as well as smart materials facilitate the release of growth factors such as bone morphogenetic proteins and vascular endothelial growth factor (VEGF), which allows enhanced MSC function and cell-mediated acceleration of tissue repair. Imaging-guided injectors and jet injectors have additionally enhanced the accuracy of MSC delivery while lowering injury to collateral tissue, further promoting therapeutic effectiveness [88].

Synergistically, MSC implants along with growth factors or gene therapies have significantly improved their regeneration capabilities. MSCs require the action of growth factors such as BMPs to efficiently induce osteogenic differentiation, thereby enhancing bone healing. Gene editing technologies such as CRISPR/Cas9 have the possibility of studying changes, enhancing the therapeutic properties of MSCs, and they can lead to targeted, personalized medicine for patients with osteoarthritis or fractures [89]. The combination of MSCs and anti-inflammatory therapy may ameliorate healing in an inflammatory bone repair model through modulation of inflammation during tissue repair. The combination therapy harnesses MSC and, in turn, EXPCC to work together, which ensures a large clinical range and enhances their regenerative therapeutic function for bone lesions and related disorders [90].

Recent studies in mesenchymal stem cell therapies have considered aspects of long-term appropriateness in terms of safety, especially the risk of tumor formation as well as an immunogenic response against allogeneic MSCs. While MSCs have been well studied in the field of bone engineering, greater success has been achieved with the use of those cell types for non-bone-related diseases including cardiovascular and neural ones, due to their robust immunomodulatory capacity [91]. Recent studies have shown that MSCs can reduce inflammation and improve tissue repair in several chronic diseases, such as diabetes and multiple sclerosis. However, unanswered questions regarding the safety and efficacy of repeated MSC administration exist, and long-term risks, including teratogenicity in vivo due to the accumulation of gene modifications from lentiviral transduction, need to be closely examined [92].

The potential improvement of outcomes in AI-based patient-data-guided personalization of dosage and delivery of MSC therapy could subsequently be followed by graduated approaches to stratify high-quality performed trials from preclinical studies. Moreover, 3D bioprinting techniques are one of the most important advances in the field of MSC delivery, because they permit localized injection of drugs that mimic the structure of bone tissue (bone-like architecture) to collaborate with osteogenesis [93]. The challenges and their possible solutions for intraosseous therapy are demonstrated in Table 3. These advancements in MSC therapies are augmented by the use of novel biomaterials coupled with sophisticated delivery systems and gene editing approaches. The ongoing and future research will continue to investigate (i) applications of MSCs in non-bone-related indications, (ii) optimal delivery methods, and (iii) the long-term safety of MSC-based therapies.

## 9. Challenges and Considerations

In the safety evaluation of MSC therapies, one major concern is tumor formation, particularly in immature MSC cell types. Preclinical and clinical assessments can reduce this risk. With accurate MSC definition and enforceable safety protocols, these risks can be minimized; for example, intraosseous delivers risks such as infection from nonsterile conditions can be minimized by strict aseptic techniques [94]. The immune rejection of allogeneic MSCs may potentially be averted via autologous MSCs or genetically targeted, thus overcoming the associated difficulties. A specific long-term safety concern, tumorigenicity, has to be further elucidated by longitudinal observations and reporting schemes [49].

The absence of standardized protocols in the preparation and administration of MSCs can limit the commonality of outcomes between clinical trials. Consequently, standardized MSC protocols are essential for effective and safe MSC therapy. Intraosseous injections are made into the main trabecular heart of the bone, and hence it is very difficult to invoke a continuous flow; optimized methods need to be developed for their preparation, transport, and monitoring. Implementation of common quality control metrics for MSCs, including functional viability and differentiation assays, is also of utmost importance [95]. The methods of delivery should aim to minimize trauma and allow for maximal retention of MSCs whilst ensuring dynamic patient monitoring and compliance with regulatory standards. To progress MSC therapies in regenerative medicine, we must overcome safety, standardization, and regulatory hurdles. Current research at the center focuses on identifying an optimal dose required for clinical effect. The therapeutic dose used in the clinical trials has indeed been highly variable and varies between target diseases, routes of administration, and study designs.

Some common strategies for dosage according to clinical studies: MSC doses used in clinical studies range typically from 1 million to 10 million cells per kilogram of body weight. For instance, studies on graft-versus-host diseases or cardiovascular diseases would employ a dose in the 1–2 million MSC/kg-per-day range. In some clinical trials, instead of body weight, the fixed dose was set for MSCs [96]. For example, studies using MSCs for the treatment of osteoarthritis and autoimmune disease used a fixed dose ranging from 50 million to 200 million cells in each administration, given as single- or repeated-dose regimes over time [97]. Many clinical studies employ repeated dosing regimens in which subjects receive multiple MSC infusions over weeks or months. For example, chronic inflammatory diseases like Crohn’s disease and chronic obstructive pulmonary disease have been treated in pilot studies by repeated dosing at an approximate 100 million cells at a dosing interval of several weeks [98].

## 10. Conclusions

Several approaches have been used to deliver mesenchymal stem cells for regenerative medicine strategies, one of which is intraosseous delivery. This strategy fine-tunes the BM microenvironment to enhance MSC homing, engraftment, and expansion. Current advancements in methods include enhancing accuracy with guided needle insertion and imaging-assisted delivery; however, combining MSCs using biomaterials and growth factors further improves regenerative outcomes. Intraosseous delivery of MSCs has potential for the treatment of orthopedic injuries and bone defects, as these cells could be nested in the supportive microenvironment represented by the BM. However, more research is needed on long-term safety and effectiveness. Even then, ethical and regulatory considerations are crucial for moving MSC therapies forward. The advancement of intraosseous MSC delivery is promising, with progress in biomaterials and personalized medicine driving the field forward. Furthermore, new biomaterials that mimic the extracellular matrix and promote MSC adhesion, survival, and differentiation could enhance therapeutic outcomes. Personalized approaches using patient-specific data, such as genetics and molecular profiles, are expected to optimize MSC therapies, improving efficacy and reducing side effects. Additionally, cell-free therapies like MSC-derived exosomes are being explored for their potential to modulate inflammation and promote tissue repair with lower risks.

## Figures and Tables

**Table 1:** Comparison of intraosseous delivery with other MSC delivery methods [56].

S. R	Condition Type	Specific Conditions	Preferred Delivery Method	Rationale for Method Choice
1	Orthopedic conditions	Fractures, osteoporosis, Raynaud’s phenomenon, carpal tunnel syndrome	Intraosseous	This targets the bone marrow directly, raising MSC retention, engraftment, and local tissue regeneration.
2	Hematological disorders	Leukemia, anemia, sickle cell disease, lymphoma, blood cancers	Intraosseous	They allow access to bone marrow where hematopoiesis occurs and lead to an improvement of the effects of MSC homing and actions in the hematopoietic environment.
3	Systemic diseases	Cardiovascular diseases, neurological diseases, respiratory inflammation, diabetes	Intravenous	Offers wide dispersal of MSCs. It is thus appropriate for systemic diseases which require therapeutically therapeutic effects.
4	Localized inflammatory conditions	Rheumatoid arthritis, localized trauma, or acute injuries	Intraosseous/local injection	Allows for better retention of MSCs in targeted areas, resulting in a targeted immune modulation and better healing effects at local sites.

**Table 2:** MSCs in orthopedic conditions, hematological disorders, and systematic diseases.

S. R	Clinical Applications	Orthopaedic Conditions	Hematological Disorders	Systemic Diseases
1	Intraosseous delivery methods as a therapeutic strategy for stimulation of MSCs	Trigger finger Repetitive strain injury Carpal tunnel syndrome Bone fracture Ganglion cyst Raynaud’s phenomenon	Leukemia Anemia Lymphoma Sickle Cell Disease Blood cancers Bleeding	Pregnancy-related issues Cardiovascular diseases Rheumatoid arthritis Neurological diseases Respiratory tract inflammation Obesity Diabetes Insulin resistance

**Table 3:** Highlights the challenges for intraosseous therapy and their possible solutions.

Challenges	Description	Possible Solutions/Continuing Research
Safety concerns	Danger and tumor formation by gene manipulation, premature aging of MSCs, infectious diseases, immune rejection.	Autologous MSCs or transplant of genetically modified cells to express immunomodulatory molecules. A tumorigenicity long-term study.
Standardization of procedures	Non-standardized protocols lead to poor reproducibility, safety and clinical outcomes. Differences in preparation, administration, and monitoring.	MSC manufacturing procedures and monitoring techniques. Designing intrathecally delivered payloads with the level of accuracy and safety required. Consistent in-process quality control for antigenic purity.
Regulatory hurdles	Concerns and a negligible approval rate, Tricky approval process with regulatory bodies such as FDA or EMA, which is being detracted by ethic practices in trials.	Enforce compliance with standard laws, regulations, and ethical standings. Clear clinical trial protocols that adhere to established guidelines.
Long-term efficacyand safety	Long-term efficacy and safety are unknown, including potential tumor genesis or other unintended side effects.	Creation of strong reporting mechanisms for adverse events, and ultimate protocols for follow-up decades out. Real-time safety and monitoring using dynamic monitoring tools.
Better delivery methods for optimization	MSC retention variability and potential for collateral trauma upon delivery.	Imaging guidance to deliver precision needle insertion. Development of delivery devices that incorporate scaffolds to aid MSC retention and engraftment.
Personalization of therapy	Patient-specific approaches to achieve optimal efficacy and minimal side effects of treatment are warranted.	Personalized medicine strategies to enable precision medicine using genetic and molecular profiling for customized therapies. Development of cell-free therapies such as MSC-derived exosomes for lessened risk.
